# Timely diagnosis of multiple endocrine neoplasia 2B by identification of intestinal ganglioneuromatosis: a case series

**DOI:** 10.1007/s12020-021-02607-2

**Published:** 2021-01-21

**Authors:** Medard F. M. van den Broek, Ester B. G. Rijks, Peter G. J. Nikkels, Victorien M. Wolters, Robert J. J. van Es, Hanneke M. van Santen, Bernadette P. M. van Nesselrooij, Menno R. Vriens, Rachel S. van Leeuwaarde, Gerlof D. Valk, Annemarie A. Verrijn Stuart

**Affiliations:** 1 Department of Endocrine Oncology, University Medical Center Utrecht, Utrecht, The Netherlands; 2 Netherlands School of Public and Occupational Health (NSPOH), Utrecht, The Netherlands; 3 Department of Pathology, University Medical Center Utrecht, Utrecht, The Netherlands; 4 Department of Pediatric Gastroenterology, Wilhelmina Children’s Hospital, University Medical Center Utrecht, Utrecht, The Netherlands; 5 Department of Oral and Maxillofacial Surgery, University Medical Center Utrecht, Utrecht, The Netherlands; 6 Department of Pediatric Endocrinology, Wilhelmina Children’s Hospital, University Medical Center Utrecht and Princess Maxima Center, Utrecht, The Netherlands; 7 Department of Medical Genetics, Wilhelmina Children’s Hospital, University Medical Center Utrecht, Utrecht, The Netherlands; 8 Department of Endocrine Surgical Oncology, University Medical Center Utrecht, Utrecht, The Netherlands; 9 Department of Pediatric Endocrinology, Wilhelmina Children’s Hospital, University Medical Center Utrecht, Utrecht, The Netherlands

**Keywords:** Multiple endocrine neoplasia 2B (MEN2B), Intestinal ganglioneuromatosis (IGN), Rectal biopsy, Neuromas/neurofibromas, Medullary thyroid carcinoma (MTC)

## Abstract

**Background:**

Medullary thyroid carcinoma (MTC) in childhood is rare and has an unfavorable prognosis. To improve outcome, early diagnosis is essential. In patients with multiple endocrine neoplasia type 2B (MEN2B), MTC can occur already before the age of 1 year. Recognition of non-endocrine features of MEN2B may lead to timely diagnosis.

**Purpose:**

To describe how early recognition of non-endocrine features can lead to a timely diagnosis of MEN2B as well as the effect of recognition of premonitory symptoms on prognosis.

**Methods:**

A retrospective case series from the University Medical Center Utrecht/Wilhelmina Children’s Hospital, a Dutch national expertise center for MEN patients. All eight MEN2B patients in follow-up between 1976 and 2020 were included and medical records reviewed.

**Results:**

Intestinal ganglioneuromatosis (IGN) as the cause of gastrointestinal (GI) symptoms was detected in seven patients. In three of them within months after birth. This led to early diagnosis of MEN2B, which allowed subsequent curative thyroid surgery. On the contrary, a MEN2B diagnosis later in childhood—in three patients (also) triggered by oral neuromas/neurofibromas—led to recurrent, persistent, and/or progressive MTC in five patients.

**Conclusions:**

Neonatal GI manifestations offer the most important window of opportunity for early detection of MEN2B. By accurate evaluation of rectal biopsies in patients with early onset severe constipation, IGN can be timely detected, while ruling out Hirschsprung’s disease. MEN2B gene analysis should follow detection of IGN and—when confirmed—should prompt possibly still curative thyroid surgery.

## Introduction

Multiple endocrine neoplasia 2B (MEN2B) is an autosomal dominant inherited cancer syndrome characterized by the co-occurrence of medullary thyroid carcinoma (MTC) in nearly 100% of patients and pheochromocytoma in 50% of patients. MEN2B differs from multiple endocrine neoplasia 2A (MEN2A) in various aspects; hyperparathyroidism occurs very rarely in MEN2B, while patients do present with numerous non-endocrine manifestations. MEN2B has an estimated prevalence of 0.9–1.7 per million, making it the rarest among the MEN syndromes [1–3]. Activating, gain-of-function germline mutations in the *REarranged Translocation* proto-oncogene (*RET* gene) were identified to cause MEN2 syndromes in the early 90s [4–6]. MEN2A is usually inherited from an affected parent, while *RET* mutations occur as de novo in 75–90% of MEN2B patients [7, 8]. The *RET* gene encodes a transmembrane tyrosine kinase receptor involved in intracellular signaling pathways of cell development required for renal organogenesis and enteric neurogenesis and is expressed in cells of the thyroid and adrenal glands, thereby explaining a part of MEN2B manifestations [9]. However, the full phenotypic spectrum of clinical manifestations associated with MEN2B has not been clarified yet.

MTC develops during the first years of life in nearly all MEN2B patients. Due to the unfavorable outcome of MTC and its early presentation, a preventive total thyroidectomy is recommended before the age of 1 year [7]. However, due to the syndrome’s rarity and frequent de novo presentation, MEN2B syndrome is not frequently recognized during early childhood. As a result, many patients already suffer from locally advanced MTC or even distant metastases when symptoms are recognized and a diagnosis of MEN2B is made [8]. Pheochromocytomas are often diagnosed in the second and third decade of life [8, 10].

Several characteristic non-endocrine manifestations are associated with MEN2B, including gastrointestinal (GI), orofacial, (musculo)skeletal, and ocular manifestations [8, 11, 12]. It is suggested that timely identification of early MEN2B manifestations can lead to early diagnosis and prevention of (incurable) MTC, thereby improving prognosis and life expectancy [8, 12, 13]. Especially non-endocrine features can play a key role in early recognition, as they might occur before inoperable MTC develops [11, 12]. MEN2B-associated diffuse intestinal ganglioneuromatosis (IGN) frequently leads to severe constipation, feeding intolerance, and/or sometimes diarrhea in the first year of life [11, 14]. Additionally, ocular symptoms, orofacial features, and musculoskeletal manifestations have been reported to occur in early childhood [11, 12]. Early recognition of these symptoms may lead to a timely diagnosis of MEN2B and (its associated) MTC.

By meticulously studying the MEN2B population in our Dutch MEN expertise center, we describe how early non-endocrine MEN2B features can lead to a timely recognition of MEN2B in clinical practice, and illustrate the effect of prompt detection on prognosis. Like a previous report from our institute, we aimed to increase awareness for these cardinal MEN2B-associated early symptoms [15].

## Materials and methods

A retrospective single-center study was conducted in the University Medical Center Utrecht (UMCU), a tertiary referral and national expertise center for pediatric and adult MEN patients. Medical records of all known MEN2B patients were reviewed from first follow-up (1976) until January 2020. Information regarding endocrine and non-endocrine disease was extracted from medical records in a standardized format. Relevant physicians’ notes and correspondence, laboratory results, imaging studies, and results from genetic analysis were taken into account.

Age-specific reference values were used to interpret laboratory data. Tumor markers used for MTC were calcitonin and carcinoembryonic antigen. Markers for pheochromocytoma were vanillylmandelic acid (VMA) (up to 2004), urine (nor)metanephrine (from 2004 until 2013), and plasma (nor)metanephrines (2013 up to 2020). *RET* mutation analysis was performed according to standard protocols (Sanger sequencing).

Thyroidectomy was performed by an experienced thyroid surgeon together with a pediatric surgeon at the age of 6 months in case of neonatal diagnosis and otherwise as soon as possible after diagnosis, in line with current international guidelines [7].

Pathological assessment of thyroid tissue was classified as normal, C-cell hyperplasia, or MTC. Rectal biopsy tissue was re-evaluated by a dedicated pathologist when the original pathology report did not provide information about the presence or absence of the MEN2B-related abnormalities (IGN).

### Definitions

Non-endocrine manifestations were reported descriptively, based upon the patients’ medical records. Periodic structural examination at non-endocrine departments (e.g., ophthalmology and oral and maxillofacial surgery) was carried out from 2007 onwards.

IGN was defined by the presence of giant ganglia combined with an increase in cholinergic nerve fibers in the submucosa of GI tissue.

Due to absence of histological diagnosis in most mucosal (oral, ocular) lesions, it was not possible to make a distinction between neuromas and neurofibromas in most cases.

Outcome of thyroid surgery regarding MTC was defined “curative” if calcitonin concentrations were undetectable postoperatively and “persistent” if still detectable post-thyroidectomy. “Recurrence of MTC” was defined as detectable calcitonin concentrations after previous curative surgery and “progressive disease” was defined as increasing calcitonin concentrations and/or evidence of metastatic disease on imaging. TNM stage was assessed using guidelines of the American Joint Committee on Cancer Cancer Staging Manual, 8th edition [16]. Diagnosis of pheochromocytoma was based on first biochemical evidence (elevated urinary VMA or urinary/plasma (nor-)metanephrines), with confirmation on imaging and pathology.

Written informed consent was obtained from parents (patients aged <12 years), patients themselves (aged ≥16 years) or both (patients aged 12–16 years). The institutional review board of the UMCU approved this study.

## Results

Eight MEN2B patients were identified (three males, five females), all carrying a de novo NM_020975.6(RET):c.2753T > C (p.Met918Thr) *RET* gene mutation. MEN2B was diagnosed at a median age of 6.3 years (range 0.1–16). Seven patients were still in follow-up at the end of the study and one had died from a metastasized pancreatic adenocarcinoma at age 54. Median clinical follow-up was 10.0 years (range 3.3–38.0). Patient characteristics are shown in Table 1.

**Table 1:** Patient characteristics and presenting symptoms of MEN2B cases

Case	Sex	Age at Dx (yr)	Follow-up time (yr)	Presenting symptom(s)	Thyroid at Dx	Pheo^a^, age at Dx (yr)
1	F	0.1	12.3	GI problems	CCH	No
2	F	0.3	7.6	GI problems	CCH	No
3	M	0.1	6.3	GI problems	MTC	No
4	M	11.7	13.8	GI problems, DMD, MW, oral NRs, CaL	MTC	Yes, 25
5	F	6.0	29.0	GI problems, DMD, dysmorphia, NRs	CCH^c^	Yes, 29^d^
6	F	15.8	6.0	Cheek NR, neck lump^b^	MTC	Yes, 21
7	F	6.5	3.3	DMD, MW	MTC	No
8	M	16.0	38.0	GR, marfanoid habitus	MTC	Yes, 16^e^

*CaL* Café au lait spot, *CCH* C-cell hyperplasia, *DMD* delayed motor development, *Dx* diagnosis, *F* female, *GI* gastrointestinal, *GR* growth retardation, *M* male, *MTC* medullary thyroid carcinoma, *MW* muscle weakness, *NR* neuroma/neurofibroma, *Pheo* pheochromocytoma, *Yr* years

^a^Anytime during follow-up. Age at first histological diagnosis of pheochromocytoma

^b^Suspicion of MEN2B because of cheek neuromas/neurofibromas, surpassed by growing neck lump

^c^Possible MTC

^d^Second primary pheochromocytoma in contralateral adrenal gland at age 33

^e^Recurrence after initial bilateral adrenalectomy at age 49

### Presenting symptoms of MEN2B

MEN2B syndrome was diagnosed solely on GI symptoms in three cases (patients 1–3) and on a combination of GI and other symptoms in two cases (patients 4–5).

**Fig. 1:**
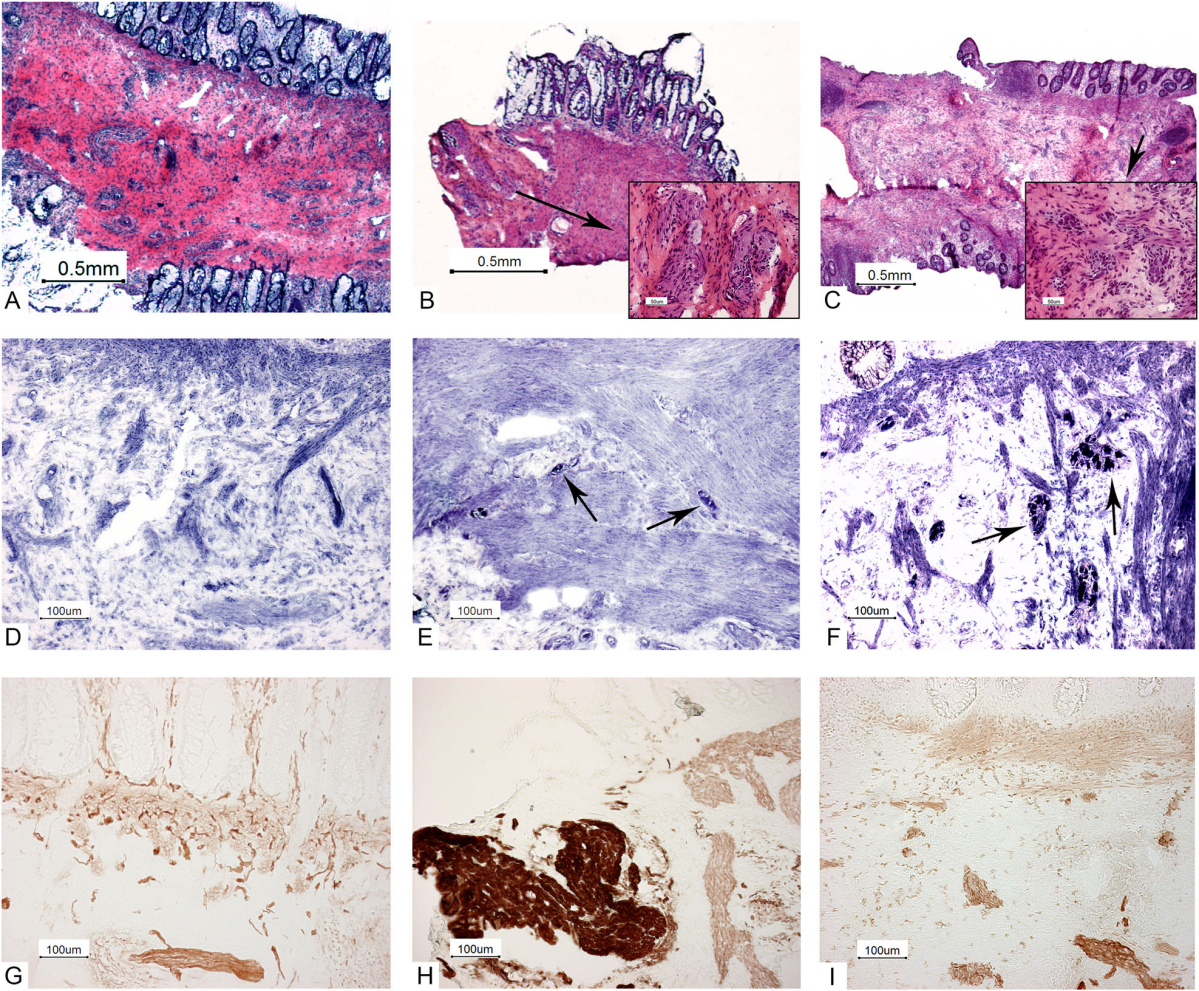
Rectal suction biopsies. Three frozen rectal suction biopsies. **A**–**C** are stained with hematoxylin and eosin (H&E). **D**–**F** are stained with NADH enzyme stain. NADH stains the cytoplasm of ganglion cells dark blue. The round nucleus of the ganglion cells does not stain and is recognizable as a white round spot in the dark blue stained cytoplasm. **G**–**I** are stained with acetylcholinesterase without counterstain. Nerve fibers stain dark yellow and the smooth muscle cells stain very weekly positive. Example patient (male 2 weeks) (left column: **A**, **D**, and **G**) with Hirschsprung’s disease: no ganglion cells present in submucosa in NADH enzyme stain (**D**). Increase in cholinergic nerve fibers (**G**) in submusosa, muscularis mucosae and in lamina propria between the crypts (upper part of the picture), characteristic for Hirschsprung’s disease. Patient 7 (girl, 6 years) (middle column: **B**, **E**, and **H**): the biopsy from this patient was very small with limited amount of submucosa and not enough for a definite diagnosis of ganglioneuromatosis but the combination of small groups of ganglion cells (inset of **B** and arrows in **E**) and broad nerve bundles (**H**) was compatible with MEN2B. Patient 3 (male, 1 month) (right column: **C**, **F**, and **I**): biopsy showed ganglioneuromatosis with a normal lamina propria and increase in ganglion cells with giant ganglia (inset of **C** and arrows in **F**) and prominent nerve bundles in the submucosa (**I**: lower part of the picture and not in the lamina propria (upper part of the picture)

The three patients diagnosed with MEN2B exclusively on GI symptoms were admitted to hospital in the first month of life for not passing stools for 5 days, increasing drowsiness and insufficient intake (patient 1), acute intestinal obstruction (patient 2) and abdominal distention, icterus and feeding difficulties (vomiting, insufficient intake) (patient 3). Patient 2 underwent a diagnostic laparotomy showing a cecal volvulus. Imaging studies in patient 3 revealed a colonic distention due to air retention. Pathological examination of rectal suction biopsies (patients 1 and 3, Fig. 1) and surgically removed tissue (patient 2) showed IGN. In all three cases, subsequent genetic analysis confirmed MEN2B diagnosis. Calcitonin level was 60 ng/l in patient 1 before surgery and unknown in patient 2 (at that time under treatment elsewhere). In patient 3, the first calcitonin level (at 3 months post-thyroidectomy) was within normal range, with later values all undetectable (see Table 2). Serum calcitonin levels have been reported to be elevated in very young children, therefore we interpreted the value of 60 ng/l in patient 1 as high but not necessarily abnormal for age, based on the report of Basuyau et al. [17].

**Table 2:** Thyroid disease in cases with MEN2B syndrome

Case	Age at surgery (yr)	Age at last FU (yr)	First available Ctn (ng/l)	Initial thyroid surgery	Histology	TNM (stage) at Dx^a^	Operation curable	Disease status at last FU
1	0.6	12.4	60^b,c^	TT	CCH	T0N0M0 (n/a)	Yes	Cured
2	0.6	7.9	U^d^	TT ± LND	CCH	T0N0M0 (n/a)	Yes	Cured
3	0.5	6.4	5^e^	TT	MTC	T1aNxMx (l)	Yes	Cured
4	12.0	25.5	360^c^	TT	MTC	T1aNxMx (l)	No	Progressive
5	6.1	35.0	0.32^c,f^	TT	CCH with possible MTC	T0N0M0 or T1aNxMx (n/a or l)	Yes	Recurrence
6	16.0	21.8	8000^c^	TT + cLND + bLND	MTC, IR	T4aN1bM1 (lVc)	No	Progressive
7	6.5	9.8	3500^c^	TT + cLND + uLND	MTC, IR	T3N1bMx (lVa)	No	Persistent
8	16.0	54.0	30^c,g^	TT ± LND	MTC	TxNxMx (?)	No	Persistent

*bLND* bilateral lymph node dissection (LND), *C* cured (no biochemical signs of thyroid disease), *CCH* C-cell hyperplasia, *cLND* central LND, *Ctn* calcitonin, *Dx* diagnosis, *FU* follow-up, *IR* irradical resection (tumor identified at the resection margin), *±LND* unknown if LND is performed, *MTC* medullary thyroid carcinoma, *n/a* not applicable, *ng/l* nanogram/liter, *P* persistent disease (biochemical signs), *Pr* progressive disease, *R* recurrent disease (biochemical signs), *TNM* tumor node metastasis—classification, *TT* total thyroidectomy, *U* undetectable, *uLND* unilateral LND, *yr* years, *?* unknown

^a^Staging based upon the American Joint Committee on Cancer (AJCC) Cancer Staging Manual, eight edition (Rosen et al. [16])

^b^Calcitonin values can be elevated in (very) young children. For reference values in children, see: Basuyau et al. [17]

^c^Preoperative calcitonin level

^d^3 years postoperative calcitonin level. Patient was under treatment in another country at time of surgery; calcitonin levels were not measured earlier

^e^3 months postoperative calcitonin level

^f^µg/l, basal ctn (normal range <0.3 µg/l)—not stimulable

^g^ng/ml, basal ctn (normal range <0.4 ng/ml). pentagastrin-stimulated ctn: 455 ng/ml (at 2 min), 310 ng/ml (at 5 min)

In patients 4 and 5, severe obstipation was present since birth as well. However, rectal suction biopsy (initially) did not raise suspicion of MEN2B in these cases. Rectal biopsy of patient 4 at 7 months showed no signs of Hirschsprung’s disease; the original pathology report did not mention the presence or absence of IGN. Unfortunately, this tissue specimen could not be retrieved for re-evaluation. Despite extensive investigations into additional symptoms (muscle weakness, delayed motor development), no explanatory diagnosis could be made at that time. Eventually, oral neuromas/neurofibromas at the age of 11 years prompted genetic analysis and the diagnosis of MEN2B syndrome. Examination of rectal tissue of patient 5 at 11 months revealed neuronal colon dysplasia without further specification. At the age of six, the combination of ongoing constipation, delayed motor development, dysmorphic features (bumpy lips, marfanoid habitus, elongated face), and histologically proven oral neurofibromas led to the diagnosis of MEN2B. Recent re-examination of the rectal biopsy tissue did, in retrospect, show clear signs of IGN.

Oral neuromas/neurofibromas were part of the presenting phenotype in three out of eight cases and were the trigger to perform genetic analysis in two (patients 4–5). Patient 6 was referred to our hospital at the age of 15 with mucosal neuromas/neurofibromas, which had been noted for several years but had not triggered suspicion of MEN2B. Before diagnostic work-up of these lesions took place, she developed a growing neck lump caused by MTC. MEN2B diagnosis was confirmed by genetic analysis soon thereafter.

Two patients in this cohort initially presented with musculoskeletal symptoms (delayed motor development, muscle weakness) (patient 7) and the combination of growth restriction and a marfanoid habitus (patient 8).

### MEN2B manifestations during follow-up

Total thyroidectomy was performed in all patients; the course of MTC is shown in Table 2. The three patients who had been diagnosed with MEN2B due to timely recognition of GI symptoms had been cured by total thyroidectomy. In total, thyroid surgery cured four out of eight patients; all were operated before the age of 6.5 years. Three other patients underwent several re-operations because of recurrent and/or progressive MTC. At last follow-up, two patients had distant metastases (patient 6: lungs and liver, patient 8: prostate).

Four out of eight patients developed pheochromocytoma during follow-up (see Table 1). Complete unilateral adrenalectomy was performed in three cases (patients 4–6), while patient 8 underwent complete bilateral adrenalectomy.

Table 3 provides an overview of all non-endocrine manifestations reported in this series during follow-up. All patients experienced GI manifestations, of which chronic obstipation with varying severity was most common. Feeding problems and obstipation since neonatal period and infancy had been present in six patients. Apart from patient 2 with neonatal volvulus and recto sigmoid resection for ileus at age 3, two additional patients required GI surgery (subtotal colectomies) for therapy-resistant obstipation at a young adult age (21 and 23 years). IGN was confirmed in seven out of eight cases: by histological examination of rectal biopsies in four patients, surgical tissue in two patients, and autopsy tissue in one patient.

**Table 3:** Non-endocrine manifestations in cases with MEN2B syndrome

Case	GI	GI—therapy	IGN (method of Dx)^a^	MSK	MBH	Oral NMs	Oral	Ocular	Other manifestations
1	+	Oral laxatives Enemas CHT SD	+ (rectal biopsy)	+	−	+	+	+	- Short stature - Transient hypogammaglobulinemia with recurrent respiratory infections - ADUS requiring meatotomy
				HL OD			CD FH	ONR A^f^ TCN	
2	+	Oral laxatives CHT Surgery	+ (surgical tissue)	?	−	−	+	+	- Short stature - Temporarily delay of growth - Anemia due to iron deficiency - Lactose intolerance
							CD FH	TCN	
3	+	Oral laxatives Enemas	+ (rectal biopsy)	+	−	+	+	−	- Short stature - Relapsing conjunctivitis
				HT			CD FH		
4	+	Oral laxatives Enemas	–^b^ (rectal biopsy)	+	+	+	+	+	- Café au lait spot cheek
				DMD MW HT HL			CD	ONR	
5	+	Oral laxatives Enemas CHT Surgery	+ (rectal biopsy)^c^	+	+	+	+	+	- Dysfunctional voiding requiring CIC
				DMD HL			CD GH FH	ONR TCN	
6	+	Oral laxatives CHT Surgery	+^d^ (surgical tissue)	+	+	+	−	+	- Café au lait spots trunk
				HL				A	
7	+	Oral laxatives Enemas	+ (rectal biopsy)^e^	+	−	+	+	+	
				DMD MW HT HL			CD FH	A	
8	+	Oral laxatives	+ (autopsy)	+	+	+	+	+	- Temporarily delay of growth - Dysfunctional voiding requiring SCAD - Kyphoscoliosis leading to dyspnea
				MW HT HL OD			GH	ONR TCN	

Non-endocrine manifestations diagnosed in cases with MEN2B patients any time during follow-up

*+* yes, − no, *A* alacrima (inability to make tears), *ADUS* anterior deflected urinary stream, *CD* central diastema, *CHT* colon hydrotherapy, *CIC* clean intermittent catheterization, *DMD* delayed motor development, *Dx* diagnosis, *FH* frenulum hyperplasia, *GH* gingiva hypertrophy, *GI* gastrointestinal, *HL* hyperlaxity, *HT* hypotonia, *IGN* intestinal ganglioneuromatosis, *MBH* marfanoid body habitus, *MSK* musculoskeletal, *MW* muscle weakness, *NMs* neuromas/neurofibromas, *OD* osseous deformities, *ONR* ocular neuromas/neurofibromas, *SCAD* continuous suprapubic catheter, *SD* manual anal internal sphincter dilatation (twice) and botulinum toxin injection into anal internal sphincter (once), *TCN* thickened corneal nerves

^a^The method of acquiring intestinal tissue (rectal biopsy, intestinal surgery, autopsy) is specified between the parentheses

^b^Rectal biopsy showed no signs of Hirschsprung’s disease. The original pathology report did not mention the presence or absence of IGN. This tissue specimen could not be retrieved for re-evaluation

^c^After recent re-examination of the tissue

^d^No rectal biopsy performed. Intestinal tissue from subtotal colectomy at the age of 21 showed IGN

^e^Biopsy after diagnosis of MEN2B

^f^Unilateral inability to make tears

In total, seven patients had been diagnosed with oral neuromas/neurofibromas. Other oral manifestations included thickened hypertrophic (bumpy) lips and maxillary midline diastema (space between central incisors). Ocular features were present in at least seven patients, including ocular neuromas/neurofibromas, prominent corneal nerves, and alacrima (the inability to cry with tears). Joint hyperlaxity—reported in six patients—was the most common musculoskeletal manifestation.

## Discussion

Timely detection of the MEN2B syndrome is only possible if the complex of symptoms is recognized. This detailed description of MEN2B cases provides insight into the non-endocrine clinical clues for diagnosis of MEN2B, before advanced or metastatic MTC develops. Although our series is small, it firstly illustrates that prevention or curation of MTC was only reached in patients in whom IGN was recognized during diagnostic work-up and thereby led to genetic analysis confirming MEN2B. Secondly, our series underlines that all patients initially presented with non-endocrine symptoms. In retrospect, MEN2B diagnosis could have been established more timely in at least two cases by proper interpretation of gastrointestinal and orofacial symptoms. Early MEN2B diagnosis was made in three out of eight patients after surgery or rectal suction biopsy for suspicion of Hirschsprung’s disease. In two other cases, oral neuromas/neurofibromas led to genetic analysis later in childhood, making this feature of MEN2B a second key element for early diagnosis.

Over the years, reports of cohorts of MEN2B patients have shown that establishing a timely diagnosis is both challenging and critical, as diagnostic delay results in worse outcome [13, 18, 19]. A median age at thyroidectomy of 14 years in the largest cohort to date (including 345 MEN2B patients) reflects the typical late diagnosis, as does the relatively small fraction of patients (20 out of 338) who were operated before the recommended age of 1 year [8]. MEN2B has been detected relatively early in life in our case series (median age at diagnosis: 6.3 year), whereas the mean age at MEN2B diagnosis reported in literature ranges from 10.6 to 18 years [13, 18, 20–22]. It is important to consider the possible effect of study period on the age at MEN2B diagnosis when comparing these results, due to the lack of DNA analysis and lower awareness for MEN2B (especially non-endocrine features) in earlier years. However, the early detection of MEN2B in our case series might also be partly explained by timely referral of young children with profound GI problems to a tertiary care hospital with both a possibility to perform rectal biopsies as well as dedicated pathologists highly aware of IGN.

Although several others have described the frequent presence of neonatal and early childhood GI symptoms in MEN2B patients [23–27], earlier studies do not focus on the clinical point we wish to make here: prevention or curation of MTC can be reached if IGN is timely recognized as the first non-endocrine manifestation of MEN2B. Severe GI symptoms in the first months of life were present in five out of eight patients in our series and IGN led to a diagnosis of MEN2B in three of them. Rectal suction biopsy is a valuable tool in diagnosing MEN2B. In this case series, IGN was reported in three out of five patients who underwent rectal biopsies (60%), while recent re-evaluation of the biopsy from patient 7 also showed IGN. As the tissue from patient 4 could not be retrieved for re-examination, we cannot rule out that the incidence of IGN in rectal biopsies may even be 100% in our series. In a literature review on GI symptoms in MEN2B patients, IGN was found in 14 out of 25 (56.0%) rectal biopsies. Furthermore, IGN was detected in 32 bowel specimens when rectal and transabdominal biopsies were combined and directly led to the diagnosis of MEN2B in 27% (15 patients), which is comparable to our findings [14]. Thus, awareness under pediatricians, pediatric gastroenterologists, pathologists, and other physicians in the field of pediatrics for IGN as a distinctive early sign of MEN2B is of great importance.

The outcomes of our case series underline previous findings on premonitory symptoms of MEN2B in larger cohorts. GI signs were, when reported, present in around two-thirds of the patients included in the international cohort by Castinetti et al. compared to 100% of patients in this case series [8]. However, differences in study setting (multicenter vs single-center), study methods, and study period make it hard to compare these results properly. In a detailed case-control study including 25 MEN2B patients, Brauckhoff et al. reported that constipation was the second most distinguishing early sign of MEN2B [11]. In a recent cohort study describing the age-related occurrence of physical stigmata in 24 MEN2B patients, gastrointestinal (and musculoskeletal) symptoms preceded symptoms of MTC significantly [12]. Likewise, the onset of GI symptoms occurred in the first year of life in 29 out of 55 MEN2B patients (53%) described in the literature review by Gfroerer et al. [14]. It was not specified whether these GI symptoms, when recognized, led to a timely (curative) thyroidectomy.

Oral neuromas/neurofibromas were the trigger to perform genetic analysis in two cases, while among the presenting symptoms in one more (out of eight cases), making it a second key element in diagnosing MEN2B. The association between mucosal neuromas/neurofibromas and MEN2B has been described earlier [28–32]. In retrospect, these manifestations had been present since childhood in most, yet unrecognized, and became more pronounced in adolescence in the majority of earlier reported cases [11, 12]. In the recently published German GPOH-MET registry study, the relatively late appearance of mucosal neuromas/neurofibromas (mean age 10.1 year) did not significantly precede symptoms of MTC [12]. However, this feature should be a trigger for further diagnostic work-up and can lead to MEN2B diagnosis [33–35]. The characteristic wide maxillary midline diastema is a non-specific feature as a midline diastema is a normal stage of dental development with a prevalence of 25–40% in children with a mixed dentition [36]. Because most children regularly visit their dentist, awareness among oral health care professionals about the typical orofacial symptoms of MEN2B should be increased [32].

Tearless crying (alacrima) is a rare sign and though possibly a feature of multiple genetic disorders [37]. It has also been reported as a potentially promising clue for timely diagnosing MEN2B: in the earlier mentioned case-control study using questionnaires, alacrima in the first year of life was reported by 86% of parents of MEN2B patients vs 0% of parents of healthy controls, making it the most distinguishing early sign for MEN2B in their study [11]. In other cohorts, alacrima was reported less frequently (17–40%) [8, 12]. This discrepancy could be explained by the different data source used in these studies (medical records), considering the potential underreporting of this symptom to the treating physicians. In our series, alacrima was present in two out of the five patients who were subjected to a structured examination at the ophthalmology department, but not part of the presenting symptoms in any of our patients. Whether alacrima has a valuable role in detecting MEN2B should be further investigated in larger prospective cohorts.

In conclusion: it is important to detect IGN in rectal biopsies even when the primary focus usually lies on the possible absence of ganglion cells, as by identification of IGN a harmful delay of diagnosis of MEN2B can be avoided. Thus, the diagnostic work-up of neonatal GI manifestations, especially severe and very early onset constipation, may create a window of opportunity for detection of MEN2B syndrome before patients suffer from locally advanced or metastasized MTC. Oral neuromas/neurofibromas in childhood may alert oral health care professionals or treating physicians for presence of the MEN2B syndrome. Large international prospective studies or databases on MEN2B patients would provide further insight into the sequence of manifestations and thus may allow early identification, ameliorating the course of MTC. Education of pediatricians, pathologists, gastroenterologists, as well as medical students, dentists and medical consultation agencies upon early identification of non-endocrine manifestations—especially gastrointestinal and oral—may help to recognize children with the MEN2B syndrome in time.

## Data Availability

The datasets generated and/or analyzed during the current study are not publicly available, but are available from the corresponding author on reasonable request.

## References

[1] Morrison PJ, Nevin NC (1996). Multiple endocrine neoplasia type 2B (mucosal neuroma syndrome, Wagenmann-Froboese syndrome). J. Med. Genet..

[2] Znaczko A, Donnelly DE, Morrison PJ (2014). Epidemiology, clinical features, and genetics of multiple endocrine neoplasia type 2B in a complete population. Oncologist.

[3] Mathiesen JS, Kroustrup JP, Vestergaard P, Madsen M, Stochholm K, Poulsen PL (2017). Incidence and prevalence of multiple endocrine neoplasia 2B in Denmark: a nationwide study. Endocr. Relat. Cancer.

[4] Mulligan LM, Kwok JBJ, Healey CS, Elsdon MJ, Eng C, Gardner E (1993). Germ-line mutations of the RET proto-oncogene in multiple endocrine neoplasia type 2A. Nature.

[5] Carlson KM, Dou S, Chi D, Scavarda N, Toshima K, Jackson CE (1994). Single missense mutation in the tyrosine kinase catalytic domain of the RET protooncogene is associated with multiple endocrine neoplasia type 2B. Proc. Natl Acad. Sci. USA.

[6] Hofstra RMW, Landsvater RM, Ceccherini I, Stulp RP, Stelwagen T, Luo Y (1994). A mutation in the RET proto-oncogene associated with multiple endocrine neoplasia type 2B and sporadic medullary thyroid carcinoma. Nature.

[7] Wells SA, Asa SL, Dralle H, Elisei R, Evans DB, Gagel RF (2015). Revised American thyroid association guidelines for the management of medullary thyroid carcinoma. Thyroid.

[8] Castinetti F, Waguespack SG, Machens A, Uchino S, Hasse-Lazar K, Sanso G (2019). Natural history, treatment, and long-term follow up of patients with multiple endocrine neoplasia type 2B: an international, multicentre, retrospective study. Lancet Diabetes Endocrinol..

[9] Norton JA, Krampitz G, Jensen RT (2015). Multiple endocrine neoplasia: genetics & clinical management. Surg. Oncol. Clin. N. Am..

[10] Thosani S, Ayala-Ramirez M, Palmer L, Hu MI, Rich T, Gagel RF (2013). The characterization of pheochromocytoma and its impact on overall survival in multiple endocrine neoplasia type 2. J. Clin. Endocrinol. Metab..

[11] Brauckhoff M, Machens A, Hess S, Lorenz K, Gimm O, Brauckhoff K (2008). Premonitory symptoms preceding metastatic medullary thyroid cancer in MEN 2B: an exploratory analysis. Surgery.

[12] Redlich A, Lessel L, Petrou A, Mier P, Vorwerk P (2019). Multiple endocrine neoplasia type 2B: frequency of physical stigmata-results of the GPOH-MET registry. Pediatr. Blood Cancer..

[13] Brauckhoff M, Machens A, Lorenz K, Bjøro T, Varhaug JE, Dralle H (2014). Surgical curability of medullary thyroid cancer in multiple endocrine neoplasia 2b: a changing perspective. Ann. Surg..

[14] Gfroerer S, Theilen TM, Fiegel H, Harter PN, Mittelbronn M, Rolle U (2017). Identification of intestinal ganglioneuromatosis leads to early diagnosis of MEN2B: role of rectal biopsy. J. Pediatr. Surg..

[15] Vasen HF, Nieuwenhuijzen Kruseman AC, Berkel H, Beukers EK, Delprat CC, van Doorn RG (1987). Multiple endocrine neoplasia syndrome type 2: the value of screening and central registration. Am. J. Med..

[16] J. Rosen, R. Lloyd, J. Brierly, Thyroid—medullary. In *AJCC Cancer Staging Manual*, 8th edn., ed. by A.B. Amid (Springer, New York, 2017), p. 891. Corrected at 4th printing

[17] Basuyau J-P, Mallet E, Leroy M, Brunelle P (2004). Reference intervals for serum calcitonin in men, women, and children. Clin. Chem..

[18] Leboulleux S, Travagli JP, Caillou B, Laplanche A, Bidart JM, Schlumberger M (2002). Medullary thyroid carcinoma as part of a multiple endocrine neoplasia type 2B syndrome: influence of the stage on the clinical course. Cancer.

[19] Raue F, Dralle H, Machens A, Bruckner T, Frank-Raue K (2018). Long-term survivorship in multiple endocrine neoplasia type 2B diagnosed before and in the new millennium. J. Clin. Endocrinol. Metab..

[20] Makri A, Akshintala S, Derse-Anthony C, Widemann B, Stratakis CA, Glod J (2018). Multiple endocrine neoplasia type 2B presents early in childhood but often is undiagnosed for years. J. Pediatr..

[21] Vasen HF, van der Feltz M, Raue F, Nieuwenhuyzen Kruseman A, Koppeschaar HP, Pieters G (1992). The natural course of multiple endocrine neoplasia type IIb. A study of 18 cases. Arch. Intern. Med..

[22] Elisei R, Matrone A, Valerio L, Molinaro E, Agate L, Bottici V (2019). Fifty years after the first description, men 2B syndrome diagnosis is still late: descriptions of two recent cases. J. Clin. Endocrinol. Metab..

[23] Carney J, Hayles A (1977). Alimentary tractmanifestations of multiple endocrine neoplasia, type 2b. Mayo Clin. Proc..

[24] Cohen MS, Phay JE, Albinson C, DeBenedetti MK, Skinner MA, Lairmore TC (2002). Gastrointestinal manifestations of multiple endocrine neoplasia type 2. Ann. Surg..

[25] Yin M, King SK, Hutson JM, Chow CW (2006). Multiple endocrine neoplasia type 2B diagnosed on suction rectal biopsy in infancy: a report of 2 cases. Pediatr. Dev. Pathol..

[26] Camacho C, Hoff A, Lindsey S, Signorini P, Valente F, Oliveira M (2008). Early diagnosis of multiple endocrine neoplasia type 2B: a challenge for physicians. Arq. Bras. Endocrinol. Metab..

[27] Gibbons D, Camilleri M, Nelson AD, Eckert D (2016). Characteristics of chronic megacolon among patients diagnosed with multiple endocrine neoplasia type 2B. U. Eur. Gastroenterol. J..

[28] Barlett R, Myall R, Bean L, Mandelstam P (1971). A neuropolyendocrine syndrome: mucosal neuromas, pheochromocytoma, and medullary thyroid carcinoma. Oral. Surg. Oral. Med. Oral. Pathol..

[29] Carney A, Sizemore G, Lovestedt S (1976). Mucosal ganglioneuromatosis, medullary thyroid carcinoma, and pheochromocytoma: multiple endocrine neoplasia, type 2b. Oral. Surg. Oral. Med Oral. Pathol..

[30] Anneroth G, Heimdahl A (1978). Syndrome of multiple mucosal neurofibromas, pheochromocytoma and medullary thyroid carcinoma. Report of a case. Int. J. Oral. Surg..

[31] O’Riordain DS, O’Brien T, Crotty TB, Gharib H, Grant CS, van Heerden JA (1995). Multiple endocrine neoplasia type 2B: more than an endocrine disorder. Surgery.

[32] Macintosh RB, Shivapuja PK, Krzemien MB, Lee M (2014). Multiple endocrine neoplasia type 2B: Maxillofacial significance in 5 cases. J. Oral. Maxillofac. Surg..

[33] Kao ST, Capua CJ, Abdelsayed RA (2018). Multiple endocrine neoplasia type 2b (MEN2B) in a 9-year-old female. J. Oral. Maxillofac. Surg..

[34] Anisowicz S, McIver H, Pedersen A (2018). Visual diagnosis: exophytic lesions on tongue and oral mucosa. Pediatr. Rev..

[35] Scott AR, Compton RA (2019). Mucosal neuromas. N. Engl. J. Med..

[36] Richardson E, Malhotra S, Henry M, Little R, Coleman H (1973). Biracial study of the maxillary midline diastema. Angle Orthod..

[37] Adams J, Schaaf CP (2018). Diagnosis and genetics of alacrima. Clin. Genet..

